# Fulminant myocarditis managed with pulsatile extracorporeal life support; use of Twin Pulse Life support (T-PLS^®^)

**DOI:** 10.1186/1749-8090-6-159

**Published:** 2011-11-24

**Authors:** Eun Jeung Cho, Joonhwa Hong, Hyun Kang, Ju Won Choe, Sang-Wook Kim

**Affiliations:** 1 Division of Cardiology, Chung-Ang University Hospital, Heukseok-dong, Dongjak-gu, Seoul, Korea 156-755; 2 Department of Thoracic and Cardiovascular Surgery, Chung-Ang University Hospital, Heukseok-dong, Dongjak-gu, Seoul, Korea 156-755; 3 Department of anesthesiology and pain medicine, Chung-Ang University Hospital, Heukseok-dong, Dongjak-gu, Seoul, Korea 156-755

**Keywords:** myocarditis, extracorporeal circulation

## Abstract

Fulminant myocarditis frequently results in severe hemodynamic deterioration. High-dose vasopressors or sometimes mechanical circulatory support are required. We report on two cases of fulminant myocarditis successfully treated with pulsatile extracorporeal life support (T-PLS^®^, Twin Pulse Life support, New heart bio.BHK, Seoul, Korea). With T-PLS, we were able to provide mechanical support to patients until they recovered completely.

## Introduction

We report on two cases of fulminant myocarditis. They developed profound hypoxemia, acidosis, pulmonary edema, and oliguria. T-PLS^®^(Twin Pulse Life support, New heart bio.BHK, Seoul, Korea) was used in order to prevent further deterioration. Both patients recovered well from fulminant myocarditis with T-PLS support. Echocardiograms showed normal ejection fraction after recovery. Written informed consent was obtained from the patient for publication of this case report and accompanying images. A copy of the written consent is available for review by the Editor-in-Chief of this journal.

### Case 1

**Figure 1:**
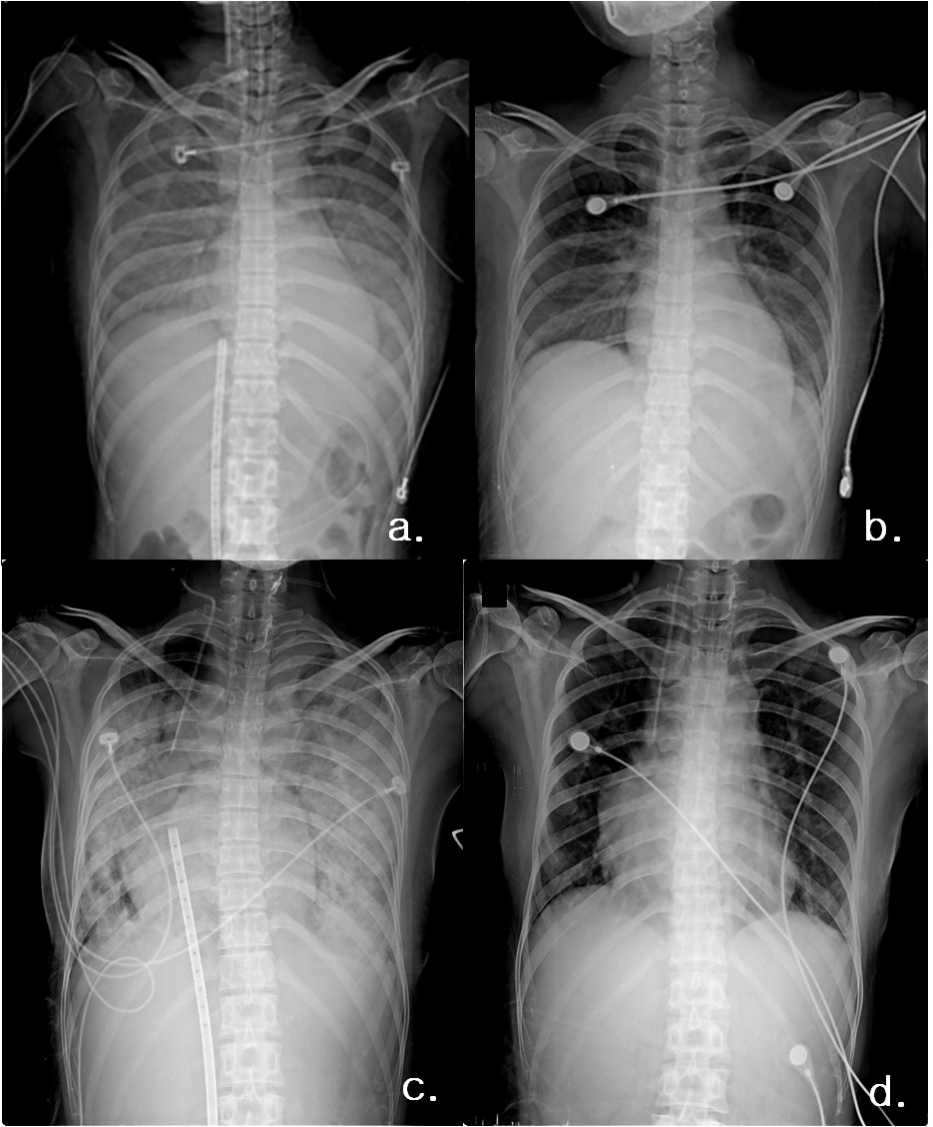
**Chest x-ray comparison of both cases**. a. Case 1, 3^rd ^day of T-PLS. b. Case 1, 10^th ^day of T-PLS, day of weaning. c. Case 2, 2^nd ^day of T-PLS. d. Case 2, 13^th ^day of T-PLS, one day after weaning.

A 32-year-old woman without previous medical history was referred to the emergency department with dizziness for one day. On admission, the vital signs were unstable, with a blood pressure of 70/40 mmHg, heart rate of 91 beats/min, and body temperature of 35.4°C. White blood cell count was 16,470 10^9^/L and troponin I was 2.563 ng/mL. Arterial blood gas analysis showed metabolic acidosis. EKG demonstrated sinus tachycardia. Hemodynamic status showed rapid deterioration during the subsequent hours. Systolic blood pressure was below 90 mmHg, with a heart rate faster than 100 beats/min. Urine output decreased, too. Hypoxemia did not resolve with supplementary oxygen. Echocardiogram showed a left ventricular ejection fraction of 21% with global hypokinesia. She was admitted to the intensive care unit (ICU) with a diagnosis of probable viral myocarditis. Ventilator therapy was initiated. She developed atrial fibrillation with rapid ventricular response. Hemodynamic status showed further worsening. Urine output further decreased and she developed hypoxemia with pulmonary edema, lactic acidosis. On the second day of admission, we decided to place the patient on a T-PLS. A 17 French femoral venous and a 14 French arterial cannula were inserted percutaneously. The pump was started with a flow of 2.13 L/min/m^2^, a pump rate of 47/min, and 100% oxygen at 4 L/min. The membrane oxygenator was replaced as required when oxygenation is not adequate (PaO_2 _< 100 with FiO_2 _of 1.0) due to plasma leakage. Serial chest X-rays showed improving pulmonary edema after T-PLS insertion (Figure 1). On day 10, T-PLS was weaned down slowly and removed. Changes of inotropic agent and pump flow rate are shown in Table 1. Blood serology showed elevated titers of Coxsackievirus B4(1:64), Coxsackievirus B2(1:16), Coxsackievirus B3.5(1:8), and Adenovirus IgM. Six days after removal of T-PLS, echocardiography showed improved cardiac function (LVEF of 68%) with minimal pericardial effusion. The patient was discharged on the 25th hospital day. Six months later, echocardiography was normal, with LVEF of 64%.

**Table 1:** Inotropics support and T-PLS setting for the initial and weaning period

		Inotropics			T-PLS settings		
		
		Norepi (μg/min)	Dopa (μg/kg/min)	Dobuta (μg/kg/min)	Flow (L/min)	Pump rate (rate/min)	Oxygen (L/min)
Case 1	Initial	30	50	15	2.13	47	4
	
	Weaning	0	4	5	1.07	33	2

Case 2	Initial	30	40	30	1.69	70	5
	
	Weaning	0	15	6	1.07	33	1.5

Norepi, Norepinephrine; Dopa, Dopamine; Dobuta, Dobutamine

### Case 2

A 36-year-old women with a history of cardiac surgery for treatment of Tetralogy of fallot 35 years ago and dextrocardia presented to the emergency department with fever and dyspnea for 2 days. On admission, blood pressure was 70/50 mmHg, pulse rate was 120 beats/min, and body temperature was 36.5°C. White blood cell count was 16,470 10^9^/L. EKG demonstrated sinus tachycardia and chest radiography showed pulmonary edema. Troponin I was elevated to 13.72 ng/mL. Echocardiogram showed depressed global left ventricular ejection fraction(28%). The patient was admitted to the intensive care unit. Nine hours after admission, troponin I increased to 42.459 ng/mL. She developed hypoxemia and hemodynamic status showed rapid deterioration with acidosis, oliguria during the subsequent hours, despite a high dose of inotropics and vasopressors. The patient was intubated and T-PLS was initiated with a percutaneous 17 French femoral venous and a 14 French arterial cannula. The pump started with a flow of 1.69 L/min/m^2^, a pump rate of 70/min, and 100% oxygen flow at 5 L/minute. Arterial blood from the right radial artery was 100% after T-PLS insertion and the patient could be extubated. The membrane oxygenator was replaced on the 3rd, 6^th^, and 9^th ^days when oxygenation was not adequate. On day 12, blood pressure was 100/60 mmHg, and pulse rate was 80 beats/min, with a T-PLS flow of 1.75 L/min/m^2^. White blood cell count decreased to 9,240 10^9^/L, and troponin I decreased to 0.130 ng/ml. Arterial blood gas analysis was satisfactory. Left ventricular systolic function (LVEF 52%) and chest x-ray (Figure 1) were much improved. T-PLS was weaned slowly down to off and removed successfully as shown in Table 1. Blood serology showed elevated titers of Coxsackie virus B4 (1:64) and Coxsackie virus B3 (1:32). Forty two days after TPLS, echocardiography showed normal systolic function (LVEF 57%) with mild pericardial effusion. Twelve months later, echocardiography showed normal cardiac function (LVEF 60%).

## Discussion

Fulminant myocarditis is an inflammation of the myocardium, which is caused by viral, bacterial, or protozoal infections, drug toxicity, or immunological reaction [1]. Fulminant myocarditis is characterized by acute onset with severe hemodynamic deterioration. Diagnosis of fulminant myocarditis in the early stages of the disease may be difficult. It may be initially misdiagnosed as septic shock, in which myocardial dysfunction and mild troponin I elevation are commonly seen [1,2]. Fulminant myocarditis is different from acute myocarditis in its clinical features [1,3]. Fulminant myocarditis was diagnosed on the basis of clinical features at presentation, including the presence of severe hemodynamic compromise, rapid onset of symptoms, and fever. On the other hand, acute myocarditis usually shows less severe hemodynamic compromise and did not have these features [3,4].

There is no specific treatment for fulminant myocarditis. Thus, treatment remains supportive. Physicians suspecting fulminant myocarditis must be prepared to use therapeutic options, such as mechanical circulatory support, prior to occurrence of severe organ failure [1,5,6]. With appropriate treatment, fulminant myocarditis is commonly reversible within a few days and is associated with better long-term prognosis than acute myocarditis [3].

Mechanical circulatory support in myocarditis is used for maintenance of cardiac output and organ perfusion, and to minimize the need for inotropic support until myocardial recovery. There are several methods for mechanical circulatory support. These include the intra-aortic balloon pump, non-pulsatile extracorporeal life support, and pulsatile extracorporeal life support, such as T-PLS, which we used for our cases, and ventricular assist devices.

A non-pulsatile pump has advantages over a pulsatile pump in that it maintains regular blood pressure with less hemolysis during total extracorporeal circulation. However, it does not maintain higher pulse pressure or mean blood pressure than the pulsatile pump [7-9], which probably results in less tissue perfusion [10].

**Figure 2:**
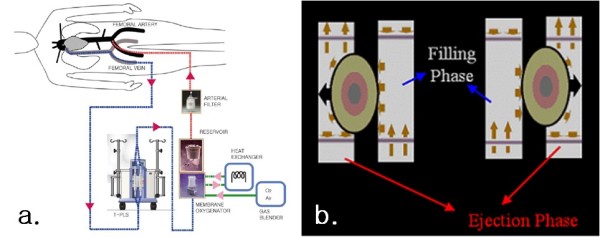
**Schematic diagram of T-PLS**. a. Connection to patient. b. Action mechanism; the reciprocating actuator pushes on blood sacs alternatively, causing pulsatile flow

T-PLS has an actuator and two blood sacs, and the reciprocating actuator pushes on the blood sacs alternatively, causing pulsatile flow (Figure 2). The T-PLS system has an effective pulsatility in hemodynamic energy and provides a beneficial effect on the coronary arteries in terms of blood flow, flow velocity, and resistance, and also provides better tissue perfusion than the non-pulsatile pump [10,11]. Compared to other pulsatile pumps with different pulse generating mechanisms, the dual sac structure of T-PLS can effectively reduce high membrane oxygenator inlet pressure, and, thus, reduce hemolysis [7,8,12,13].

T-PLS is not synchronized with native heart beat. When it is used in severe heart failure patient where there is very low pulsatility from the heart, no synchronization is required. When the heart is recovered and makes higher pulsatility, T-PLS rate is weaned down so that it does not eject too strongly when the heart ejects.

As proven by previous studies, T-PLS can improve coronary and other tissue perfusion with less hemolysis [7,8,12], and those advantages are essential for treatment of fulminant myocarditis; therefore, we think that T-PLS is one of the most suitable treatment options for fulminant myocarditis.

## Competing interests

The authors declare that they have no competing interests.

## Authors' contributions

EC, JH and SK participated in the sequence alignment and drafted the manuscript. JC participated in taking care of the patients. HK participated in the image preparation and the patients' medical record review. All authors read and approved the final manuscript.

## References

[B1] Burian J Buser P Eriksson U Myocarditis; the immunologist's view on pathogenesis and treatment Swiss med wkly 2005 135 359 364 16106325 10.4414/smw.2005.10940

[B2] Montcriol A Wiramus S Ribeiri A Successful management of influenza A associated fulminant myocarditis; mobile circulatory support in intensive care unit; a case report Case J 2008 1 46 10.1186/1757-1626-1-46 PMC2494987 18638393

[B3] Felker GM Boehmer JP Hruban RH Echocardiographic Findings in Fulminant and Acute Myocarditis J Am Coll Cardiol 2003 36 227 232 10.1016/s0735-1097(00)00690-2 10898439

[B4] Geluk CA Otterspoor IC Boeck B Magnetic resonance imaging in acute myocarditis; a case report and a review of literature The Netherlands journal of Med 2002 60 5 223 227 12365479

[B5] Sasaki H Kawai A Kuosawa H Mechanical Support for Patients with Fulminant Acute Myocarditis: Strategy for Biventricular Failure and Respiratory Failure J Card Surg 2008 23 526 527 10.1111/j.1540-8191.2008.00602.x 18482392

[B6] Shin JS Lee SW Han GS Successful extracorporeal life support in cardiac arrest with recurrent ventricular fibrillation unresponsive to standard cardiopulmonary resuscitation Resuscitation 2007 73 309 313 10.1016/j.resuscitation.2006.09.011 17257730

[B7] Lee HS Rho YR Lee HS In vivo evaluation of the pulsatile ECLS system J Artif Organs 2003 6 1 25 29 10.1007/s100470300004 14598121

[B8] Rho YR Choi H Lee JC Applications of the pulsatile flow versatile ECLS; in vivo studies Int J Artif Organs 2003 26 428 435 12828310 10.1177/039139880302600509

[B9] Lee DH Jung TE Lee JH Extracorporeal Life Support with a twin-pulse Life Support (T-PLS) System Korean J Thorac cardiovasc Surg 2007 40 512 516

[B10] Lee HS Rho YR Lee HS In vivo evaluation of the pulsatile ECLS system J Artif Organs 2003 6 25 29 10.1007/s100470300004 14598121

[B11] Kim HK Son HS Fanc YH The Effects of Pulsatile Flow Upon Renal Tissue Perfusion During Cardiopulmonary Bypass: A Comparative Study of Pulsatile and Nonpulsatile Flow ASAIO J 2005 51 30 36 10.1097/01.MAT.0000150324.02040.B4 15745131

[B12] Lee JJ Lim CH Son HS In vitro evaluation of the performance of korean pulsatile ECLS(T-PLS) Using precise Quantification of pressure-Flow Waveforms ASAIO J 2005 51 604 608 10.1097/01.mat.0000176240.78374.16 16322725

[B13] Lim CH Son HS Lee JJ Optimization of the circuit configuration of a pulsatile ECLS, An In Vivo Experimental study ASAIO J 2005 51 609 613 10.1097/01.mat.0000177779.59381.95 16322726

